# Comparison of Protein Binding and Fluorescence Quenching by Gold and Iron Oxide Nanoparticles: pH and Temperature Dependence

**DOI:** 10.3390/molecules31122008

**Published:** 2026-06-08

**Authors:** Elena A. Molkova, Ruslan M. Sarimov, Tatyana A. Matveeva, Alexander V. Simakin, Arthur G. Akopdzhanov, Philipp Sharafullin, Polina Pichkur, Aleksey S. Dorokhov, Andrey Yu. Izmaylov, Sergey V. Gudkov

**Affiliations:** 1 Prokhorov General Physics Institute of the Russian Academy of Sciences, 119991 Moscow, Russia; bronkos627@gmail.com (E.A.M.); rusa@kapella.gpi.ru (R.M.S.); pticek@yandex.ru (T.A.M.); avsimakin@gmail.com (A.V.S.); filipp.sharafullin@mail.ru (P.S.); 3602061@gmail.com (P.P.); 2 Russian National Pirogov Research Medical University, 117997 Moscow, Russia; artura777@mail.ru; 3 Department of Fundamental Sciences, Bauman Moscow State Technical University, 5, 2nd Baumanskaya St., 105005 Moscow, Russia; 4 Federal Scientific Agroengineering Center VIM, 109428 Moscow, Russia; dorokhov.vim@yandex.ru (A.S.D.); vim@vim.ru (A.Y.I.)

**Keywords:** protein nanoparticle interaction, binding constant, fluorescence quenching, pH, temperature, gold nanoparticles, iron oxide nanoparticles

## Abstract

Analysis of protein binding affinity to nanoparticles is essential for understanding how nanoparticles behave in biological systems and for optimizing their applications in medicine and biotechnology. This study demonstrates the dependence of protein binding and fluorescence quenching constants (HEWL and BSA) in the presence of gold (AuNP) or iron oxide (IONP) nanoparticles on pH and temperature. The highest binding and quenching constants were observed for proteins with gold nanoparticles (~10^9^ M^−1^). No clear effect of pH or temperature on either the binding or quenching constants of proteins with gold nanoparticles was detected. Conversely, different temperature trends were observed for the binding and quenching constants at different pH levels and for different proteins with iron oxide nanoparticles. It was shown that the nature of the nanoparticles has the strongest influence on their interactions with proteins, while the influence of environmental conditions can be considered secondary.

## 1. Introduction

Gold and iron oxide nanoparticles serve as promising platforms for application in medicine and biotechnology [1,2,3]. In particular, iron oxide nanoparticles are used in hyperthermia therapy [4], tissue imaging [5], drug delivery [6], and biosensors [7]. Gold nanoparticles are promising for use as a contrast agent in CT [8] or in optical imaging instead of fluorophores [9]. The efficiency of using nanoparticles in biological systems significantly depends on their interaction with plasma and tissue proteins, which leads to the formation of a biomolecular “corona” [10] and sometimes the formation of aggregates [11]. The protein corona significantly changes the properties of nanoparticles and affects their stability [12]. Numerous studies show that the formation of a protein corona reduces the cytotoxicity of nanoparticles [13,14,15]. Nanoparticles with a “corona” have differences in adhesion and internalization into cells compared to uncoated nanoparticles [16]. The interaction of nanoparticles with proteins can significantly alter biological effects at the cellular level [17].

The affinity of proteins with nanoparticles can be characterized using various physical parameters and constants, including the binding constant [18,19] or the quenching constant [20,21]. The binding constant is mainly determined using optical methods (absorption and fluorescence spectroscopy) or differential scanning calorimetry [22,23]. The quenching constant is determined using fluorescence spectroscopy. Upon complex formation, nanoparticles attenuate the intensity of emitted fluorescence from the protein due to Förster resonance energy transfer; therefore, the degree of quenching largely depends on the distance between the aromatic residues and the nanoparticle surface [24]. Conformational changes in the protein molecule, as well as the formation of aggregates upon the addition of nanoparticles, can lead to changes in fluorescence intensity [25]. Temperature and pH of the environment have a significant impact on the binding of nanoparticles to proteins [26,27,28,29,30]. For example, the study demonstrated the strongest interaction of lysozyme with nanoparticles at pH 9.0 compared to pH 7.4 between lysozyme and functionalized iron oxide nanoparticles [31]. Significant changes occurred in the structure of both BSA and HSA upon interaction with silicon nanoparticles with an increase in temperature from 25 to 37 °C [32]. Determining the interaction constants between proteins and nanoparticles allows us to expand understanding of the formation of nanoparticle–protein complexes under different pH and temperature conditions, including for predicting the pharmacokinetic parameters of nanoparticles [33]. The study presents the results of a study of the influence of pH and temperature on the binding and quenching constants during the interaction of gold and iron oxide nanoparticles with proteins.

## 2. Results

### 2.1. Characterization of Gold and Iron Oxide Nanoparticles

**Figure 1:**
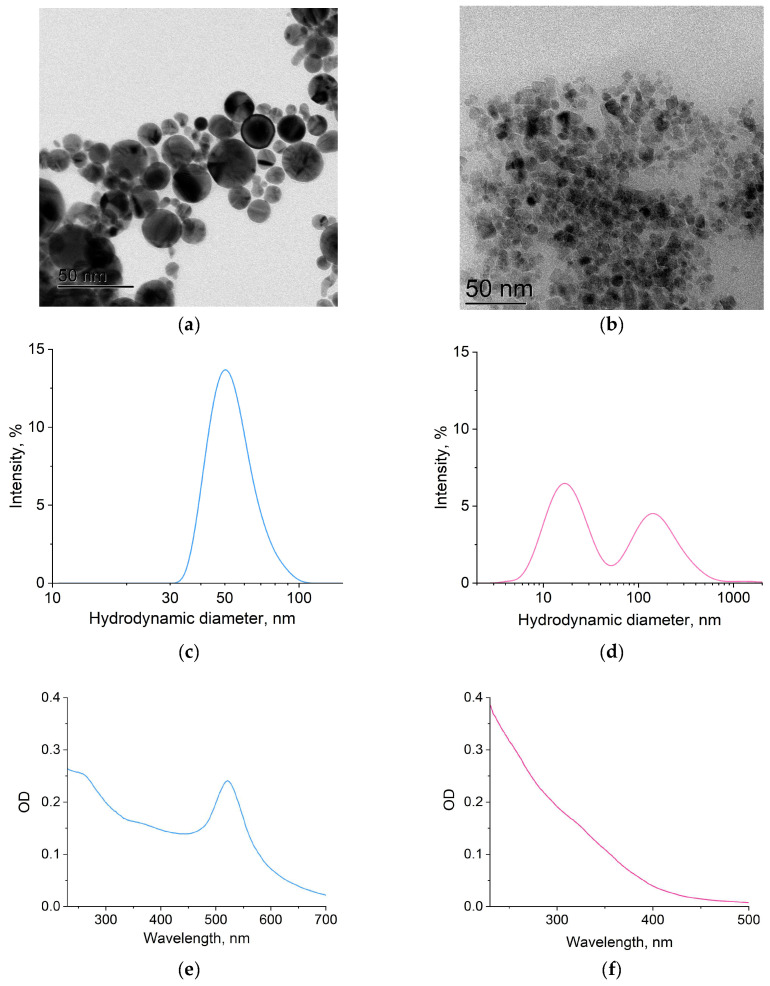
Size characteristics of gold and iron oxide nanoparticles. TEM images of dry gold (**a**) and iron oxide (**b**) nanoparticles. Distribution of hydrodynamic diameters of gold (**c**) and iron oxide (**d**) nanoparticles in an aqueous colloid. Absorption spectra of 0.50 nM gold (**e**) and 0.83 nM iron oxide (**f**) nanoparticles in an aqueous colloid.

The resulting microscopic images of gold and iron oxide nanoparticles are shown in Figure 1a,b. The median size of gold nanoparticles in vacuum was 15–35 nm (Figure 1a), while that of iron oxide was 5–10 nm (Figure 1b). Dynamic light scattering showed that the hydrodynamic diameter of the nanoparticles in liquid is slightly larger: 50 nm for gold nanoparticles (Figure 1c) and 15 nm for iron oxide nanoparticles (Figure 1d). In addition, the distribution of hydrodynamic diameters of iron oxide nanoparticles contains a fraction of approximately 150 nm in size (Figure 1d). Nanoparticles of this size were not observed using electron microscopy. Clusters of several iron oxide nanoparticles likely form in the liquid. XRD analysis was also performed on the iron oxide nanoparticles. It shows that the nanoparticles are predominantly composed of Fe_3_O_4_/γ-Fe_2_O_3_ (Appendix A, Figure A9).

### 2.2. Determination of the Binding Constant of Proteins to Nanoparticles

**Figure 2:**
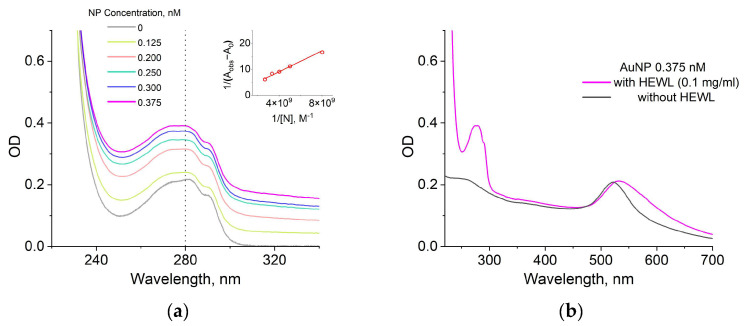
Absorption spectra of lysozyme in the presence of gold nanoparticles at concentrations of 0, 0.125, 0.2, 0.25, 0.3, 0.375 nM at 25 °C and pH 5.0 (**a**). The reduced image in the upper right corner shows the Benesi–Hildebrand plot, constructed based on the absorption spectra data. Absorption spectra of gold nanoparticles (0.375 nM) in the presence and absence of HEWL (0.1 mg/mL) at a 25 °C and a pH of 5.0 (**b**).

The Benesi–Hildebrand method is an approach used for the determination of the association constant between substances. For determination of the binding constant by the Benesi–Hildebrand method, the absorption at a wavelength of 280 nm was measured of aqueous colloids of lysozyme or BSA proteins (0.1 mg/mL) with gold nanoparticles (at concentrations from 0.125 nM to 0.375 nM) or iron oxide nanoparticles (at concentrations from 0.83 nM to 8.3 nM) at temperatures of 25, 45, 65 and 85 °C and pH values of 2.0, 5.0, 7.5 and 12.0. As an example, Figure 2a shows the absorption spectra of lysozyme protein in the presence of gold nanoparticles in different concentrations at room temperature and pH 5.0. The absorption of the protein colloid without nanoparticles (gray curve) was taken as A0. The thumbnail in the upper right corner shows a Benesi–Hildebrand plot constructed from the absorbance values of protein colloid solutions with different nanoparticle concentrations. The absorbance of the solutions at 280 nm was substituted for Aobs to calculate the values on the ordinate axis. It is worth noting that the increase in the absorption of protein colloids with nanoparticles is due to the dispersion of nanoparticles, and not the formation of aggregates (Figure 2b).

**Figure A1:**
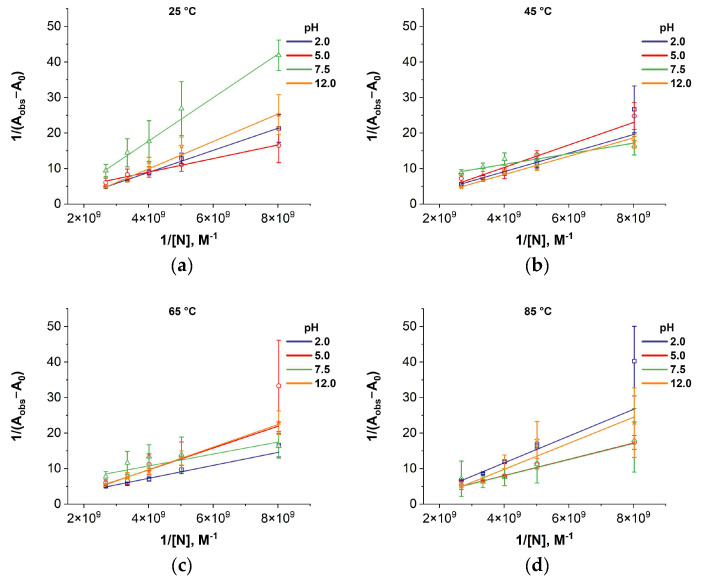
Benesi–Hildebrand plots for colloid containing lysozyme together with gold nanoparticles at pH 2.0, 5.0, 7.5, and 12.0 and temperatures of 25 °C (**a**), 45 °C (**b**), 65 °C (**c**), and 85 °C (**d**). Absorption was measured at a wavelength of 280 nm, averaging over three measurements.

**Figure A2:**
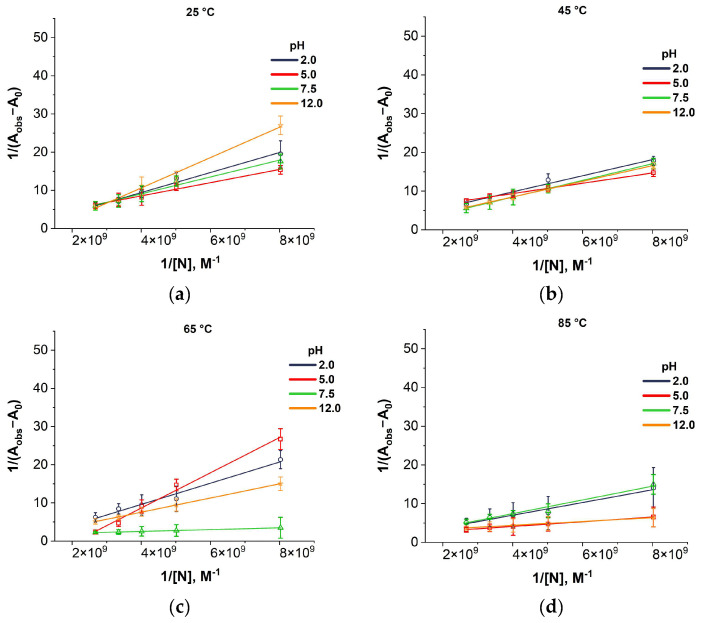
Benesi–Hildebrand plots for colloid containing BSA together with gold nanoparticles at pH 2.0, 5.0, 7.5, and 12.0 and temperatures of 25 °C (**a**), 45 °C (**b**), 65 °C (**c**), and 85 °C (**d**). Absorption was measured at a wavelength of 280 nm, averaging over three measurements.

**Figure A3:**
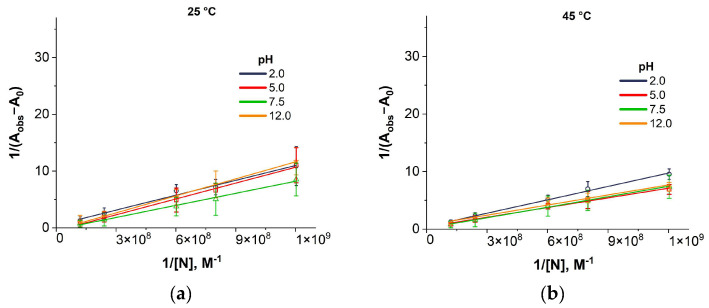

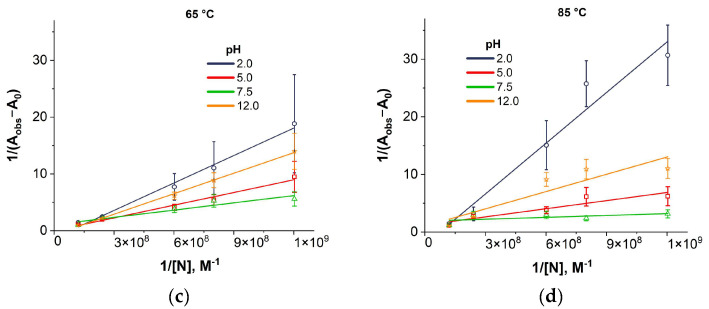
Benesi–Hildebrand plots for colloid containing lysozyme together with iron oxide nanoparticles at pH 2.0, 5.0, 7.5, and 12.0 and temperatures of 25 °C (**a**), 45 °C (**b**), 65 °C (**c**), and 85 °C (**d**). Absorption was measured at a wavelength of 280 nm, averaging over three measurements.

**Figure A4:**
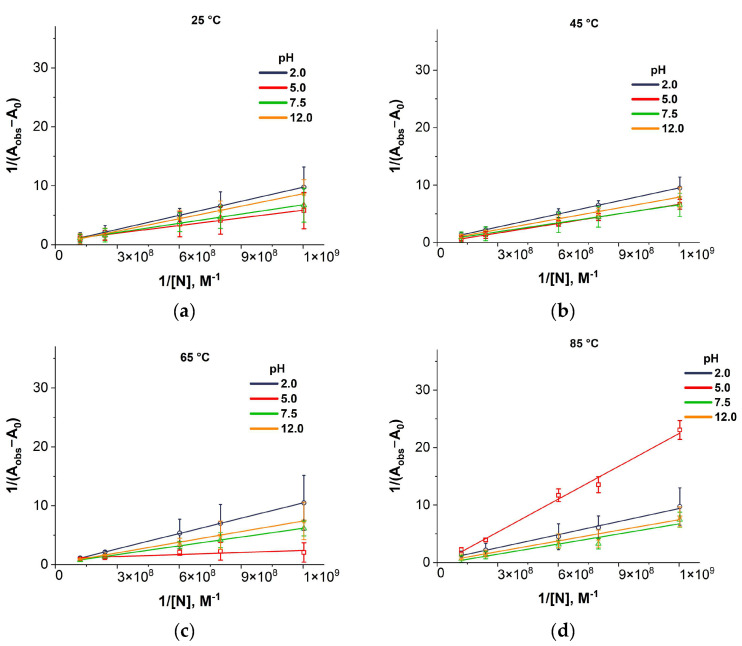
Benesi–Hildebrand plots for colloid containing BSA together with iron oxide nanoparticles at pH 2.0, 5.0, 7.5, and 12.0 and temperatures of 25 °C (**a**), 45 °C (**b**), 65 °C (**c**), and 85 °C (**d**). Absorption was measured at a wavelength of 280 nm, averaging over three measurements.

Figure A1, Figure A2, Figure A3 and Figure A4 (Appendix A) show the Benesi–Hildebrand plots for four cases of gold/iron oxide nanoparticle interactions with HEWL/BSA proteins at different temperatures and pH. Linear fitting was performed using the least-squares method. The binding constant was determined from Equation (5) as the ratio of the y-intercept to the slope of the trend line in each case (Table A1). Further, all binding constants were determined in a similar manner.

**Figure 3:**
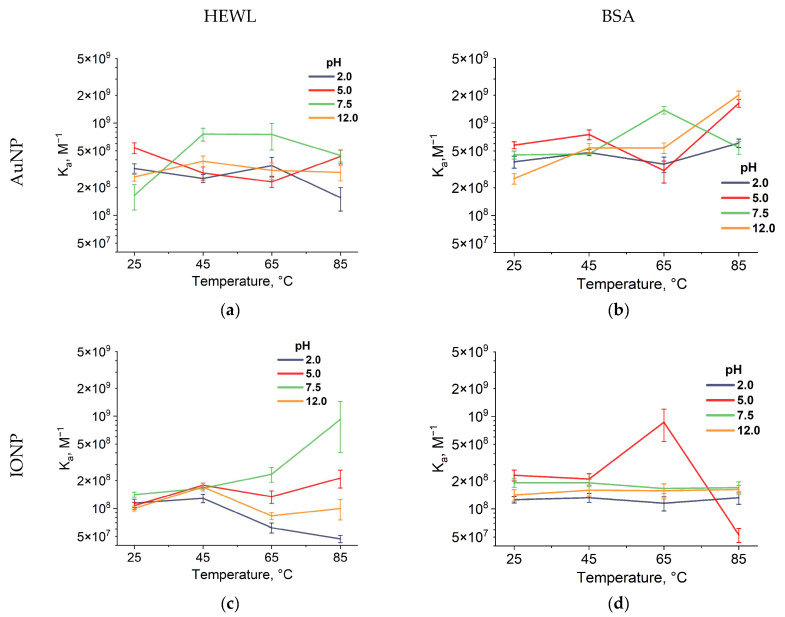
Temperature dependence of protein association constants with nanoparticles at pH 2.0, 5.0, 7.5, and 12.0. Temperature dependence of lysozyme association constants with gold nanoparticles (**a**) and iron oxide nanoparticles (**c**); BSA with gold nanoparticles (**b**) and iron oxide nanoparticles (**d**). Each point represents the average of three independent measurements.

Figure 3 shows the dependences of the binding constants of the two studied proteins, HEWL and BSA, on AuNPs and IONPs (obtained from B-H plots (Figure A1, Figure A2, Figure A3 and Figure A4, Appendix A). In general, for most pH values, the binding constants of HEWL to AuNPs do not change significantly with temperature and lie in the range of 1.5–7.6 × 10^8^ M^−1^ (Figure 3a). However, at pH 7.5, an increase in K_a_ to 7.5 × 10^8^ M^−1^ at 65 °C is observed. A similar situation is observed for the association constant of AuNPs with BSA (an increase to 14 × 10^8^ M^−1^ at 65 °C, Figure 3b). For Ka at pH 5.0 and 12.0, an increase to 16–20 × 10^8^ M^−1^ occurred at a temperature of 85 °C (Figure 3b).

For IONPs, the association constant is strongly dependent on pH and temperature (Figure 3c,d). When HEWL interacts with IONPs under acidic conditions (pH 2.0), the Ka values decrease to 0.47 × 10^8^ M^−1^ with increasing temperature (Figure 3c). A similar trend for HEWL in an acidic medium and high temperature was observed during interaction with AuNPs (Figure 3a). A noticeable increase in K_a_ to 9.2 × 10^8^ M^−1^ with increasing temperature was observed at pH 7.5. All binding constants of BSA with IONPs lie in a narrow range of values from 1.5 to 2.3 × 10^8^ M^−1^ (Figure 3d). The only exception is observed at pH 5.0, where with increasing temperature, Ka first increases to 8.7 × 10^8^ M^−1^ and then decreases to 0.5 × 10^8^ M^−1^.

Since electrostatic forces make the greatest contribution to the interaction of nanoparticles and proteins [34], the electrokinetic potential of solutions was measured at 25 °C (Table 1) and different pH. In general, an increase in temperature did not lead to a change in the electrokinetic potential in colloids. Therefore, the electrokinetic potentials of some solutions were selectively measured at 65 °C. In a BSA solution at pH 5.0, an electrokinetic potential close to zero (−2.7 ± 0.8 mV) was observed, while the binding constants for this protein were highest at 25 °C. The value of the electrokinetic potential in an aqueous BSA colloid changes slightly in the presence of gold nanoparticles and is +5.0 ± 1.2 mV at 25 °C, and does not change significantly at +5.2 ± 2.1 mV at 65 °C. Moreover, for a colloid containing both BSA and IONP at pH 5.0, a decrease in electrokinetic potential from −2.5 ± 1.0 to −8.4 ± 1.4 mV is observed with an increase in temperature from 25 °C to 65 °C.

The highest binding constant of lysozyme to gold nanoparticles was observed at pH 5.0 and 25 °C. Under these conditions, the protein is most stable in solution and likely has the most favorable conformation for binding to gold nanoparticles. Also, positively charged lysozyme (+16 ± 0.3 mV) can be effectively attracted to the negatively charged surface of nanoparticles (−26 ± 2.4 mV). However, the highest binding constant was observed at pH 7.5 in the case of lysozyme with iron oxide nanoparticles. It was in this case that a change in the electrokinetic potential was observed from +10 ± 0.7 to +25.2 ± 1.5 mV with an increase in temperature from 25 to 65 °C.

Overall, the highest binding constants were observed for protein–AuNP interactions across the entire pH range. No significant effect of temperature on binding constants was observed. In contrast, a significant effect of temperature on protein–IONP interactions was observed at different pH levels.

### 2.3. Determination of the Quenching Constant of Protein Fluorescence by Nanoparticles

**Figure 4:**
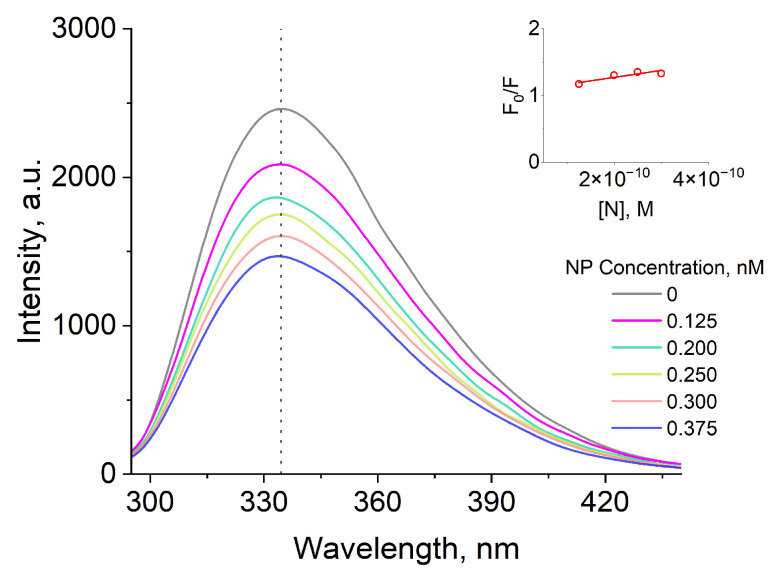
Fluorescence spectra of lysozyme in the presence of gold nanoparticles at concentrations of 0, 0.125, 0.2, 0.25, 0.3, and 0.375 nM at 25 °C and pH 5.0. The reduced image in the upper right corner shows the Stern–Volmer plot constructed based on the fluorescence spectra data.

To determine the quenching constant by the Stern–Volmer method, the fluorescence spectra of lysozyme or BSA (0.1 mg/mL) proteins were measured during their interaction with gold nanoparticles (at concentrations from 0.125 nM to 0.375 nM) or iron oxide nanoparticles (at concentrations from 0.83 nM to 8.3 nM) at temperatures of 25, 45, 65 and 85 °C and pH values of 2.0, 5.0, 7.5 and 12.0. Excitation occurred at a wavelength of 280 nm. As an example, Figure 4 shows the fluorescence spectra of the lysozyme protein in the presence of AuNPs of different concentrations at room temperature and pH 5.0. F_0_ was taken as the fluorescence of the protein without nanoparticles (gray curve). The thumbnail in the upper right corner shows a Stern–Volmer plot constructed based on the obtained protein fluorescence values with different nanoparticle concentrations.

**Figure 5:**
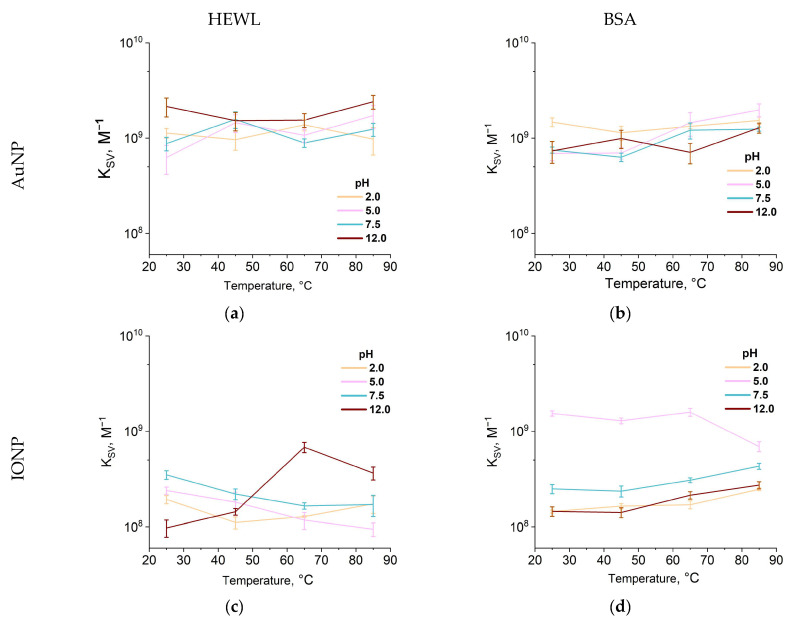
Temperature dependences of protein fluorescence quenching constants in the presence of nanoparticles at pH values of 2.0, 5.0, 7.5, and 12.0. For the interaction of lysozyme molecules with gold nanoparticles (**a**) or iron oxide nanoparticles (**c**); and BSA molecules with gold nanoparticles (**b**) and iron oxide nanoparticles (**d**). Each point represents the average of three independent measurements.

**Figure A5:**
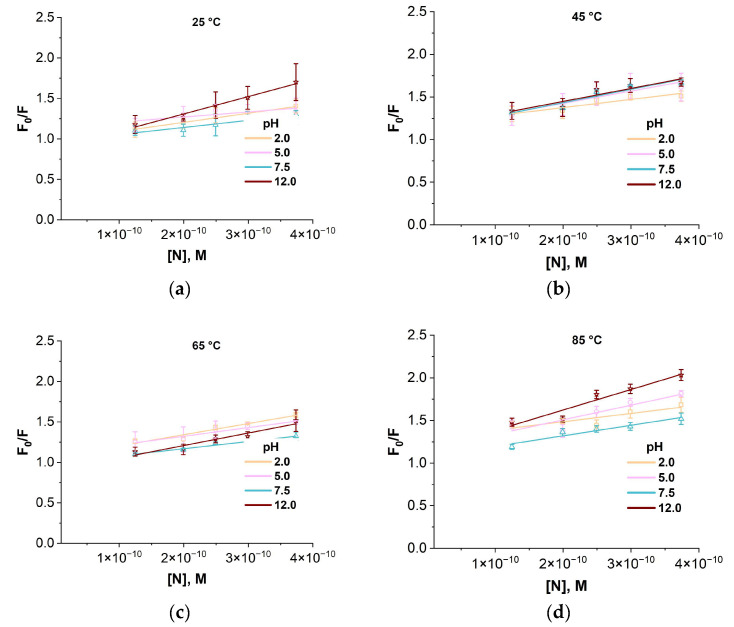
Stern–Volmer fluorescence quenching plots for colloid containing lysozyme together with gold nanoparticles at pH 2.0, 5.0, 7.5, and 12.0 and temperatures of 25 °C (**a**), 45 °C (**b**), 65 °C (**c**), and 85 °C (**d**). Fluorescence was measured at an excitation wavelength of 280 nm. Averaging over three measurements.

**Figure A6:**
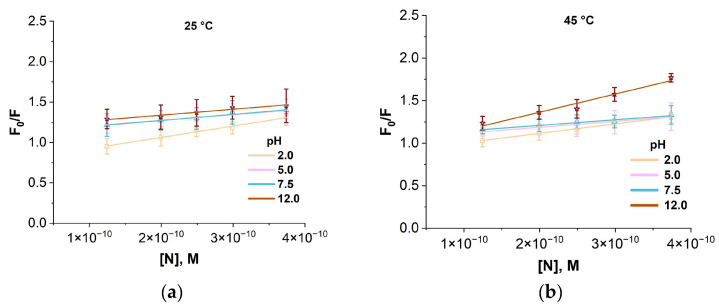

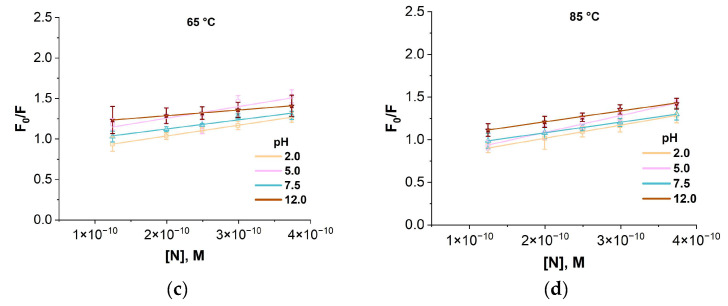
Stern–Volmer fluorescence quenching plots for colloid containing BSA together with gold nanoparticles at pH 2.0, 5.0, 7.5, and 12.0 and temperatures of 25 °C (**a**), 45 °C (**b**), 65 °C (**c**), and 85 °C (**d**). Fluorescence was measured at an excitation wavelength of 280 nm. Averaging over three measurements.

**Figure A7:**
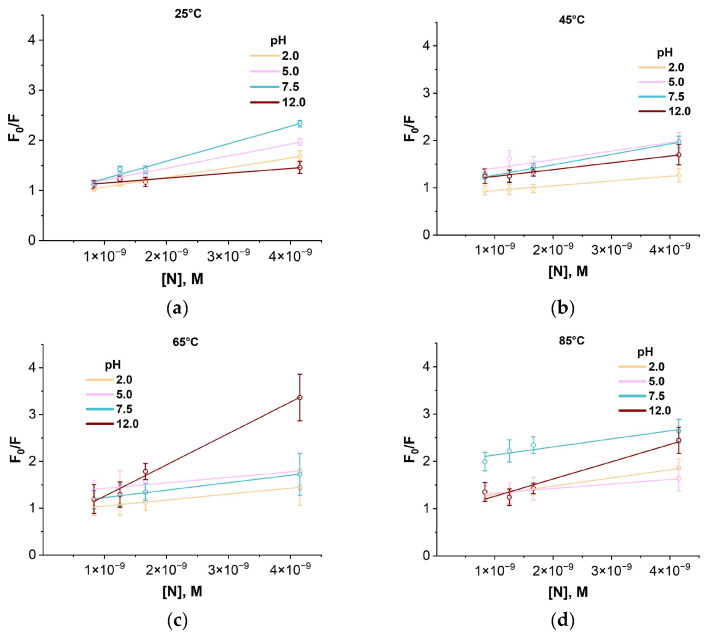
Stern–Volmer fluorescence quenching plots for a colloid containing lysozyme together with iron oxide nanoparticles at pH 2.0, 5.0, 7.5, and 12.0 and temperatures of 25 °C (**a**), 45 °C (**b**), 65 °C (**c**), and 85 °C (**d**). Fluorescence was measured at an excitation wavelength of 280 nm. Averaging over three measurements.

**Figure A8:**
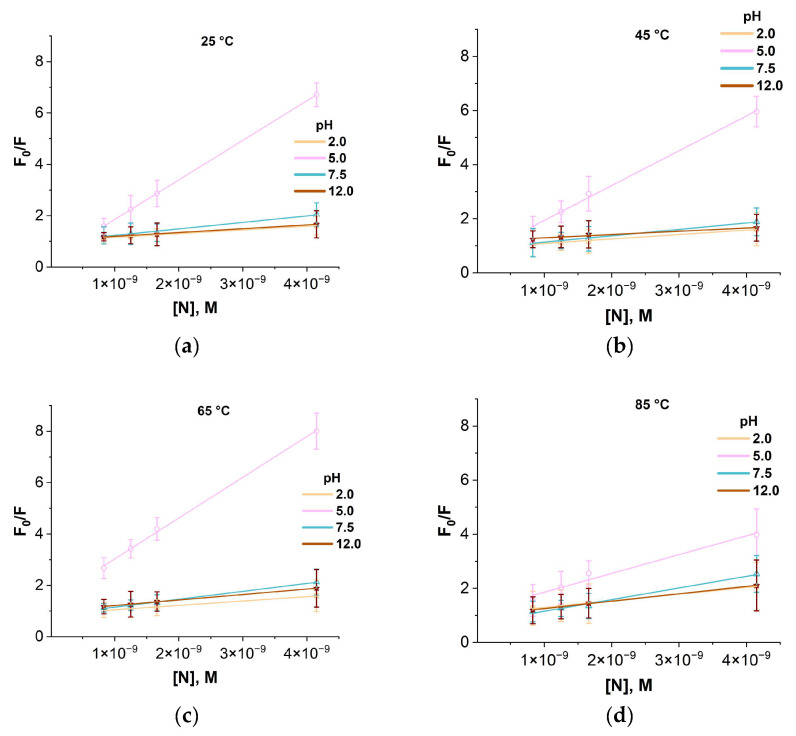
Stern–Volmer fluorescence quenching plots for colloid containing BSA together with iron oxide nanoparticles at pH 2.0, 5.0, 7.5, and 12.0 and temperatures of 25 °C (**a**), 45 °C (**b**), 65 °C (**c**), and 85 °C (**d**). Fluorescence was measured at an excitation wavelength of 280 nm. Averaging over three measurements.

Figure 5 shows the dependences of the fluorescence quenching constants of the two studied proteins, HEWL and BSA, on AuNPs and IONPs (obtained from the graphs in Figure A5, Figure A6, Figure A7 and Figure A8, Appendix A). In general, when interacting with AuNPs, it is difficult to observe trends in the quenching constant as a function of temperature at different pH (Figure 5a,b). The greatest fluorescence quenching of HEWL in the presence of AuNPs occurs at pH 12.0 and 25 °C with a quenching constant of 21 × 10^8^ M^−1^ (Figure 5a). A tendency for the quenching constant to increase for BSA to 12 and 20 × 10^8^ M^−1^ with increasing temperature can also be noted at pH 5.0 and 7.5, respectively (Figure 5b).

No statistically significant changes in the quenching constant are observed in the interaction of gold nanoparticles with lysozyme or BSA proteins (Figure 5a,b). In the case of protein interaction with IONP, changes in pH and temperature have a significant effect on the quenching constant. Only at pH 2, no statistically significant changes in the quenching constant are observed in the interaction of HEWL with IONP. At pH 5 to 7.5, a trend toward a decrease in the quenching constant from 2.4–3.5 × 10^8^ M^−1^ to 0.9–1.7 × 10^8^ M^−1^ is observed in the interaction of HEWL with IONP (Figure 5c). The lowest quenching constant of 0.98 × 10^8^ M^−1^ was observed at pH 12.0 and a temperature of 25 °C. A nonlinear change in the quenching constant was observed with increasing temperature at pH 12. Up to 65 °C, the quenching constant increases to 6.8 × 10^8^ M^−1^, while at 85 °C, it decreases slightly to 3.7 × 10^8^ M^−1^. When BSA interacts with IONPs at pH values of 2, 5, and 12, the quenching constant tends to increase to 2.5–4.3 × 10^8^ M^−1^ (Figure 5d). At pH 5.0 and temperatures of 25–65 °C, the highest quenching constants are observed for BSA-IONPs (1.3–1.6 × 10^9^ M^−1^) (Figure 5d). As the temperature increases to 85 °C, the constant decreases to 6.9 × 10^8^ M^−1^. Overall, the protein quenching constant upon the addition of gold nanoparticles depends weakly on pH and temperature. In contrast, the quenching constants for proteins in the presence of iron oxide nanoparticles change with temperature and pH. In this case, different temperature trends were observed for different proteins. Furthermore, at certain pH values, the temperature trend reversed at 85 °C.

## 3. Discussion

According to the literature, protein adsorption occurs due to the interaction of the nanoparticle surface with the amino acid residues of the protein. Hydrophilic amino acids such as glutamic acid and lysine have a high affinity for the surface of iron oxides, whereas the hydrophobicity of valine, proline, and tyrosine can limit their adsorption [35]. In addition, the presence of available thiol groups (e.g., cysteine) dramatically increases the affinity of the protein for gold nanoparticles due to the formation of strong covalent bonds Au-S. Thus, in [36], a high affinity for the surface of HSA and Cytochrome C proteins is reported due to the free thiol. The structure of BSA contains one free thiol group Cys-34 [37], unlike lysozyme, in the structure of which 8 thiol groups form 4 disulfide bridges [38]. Accordingly, the highest binding constants of gold nanoparticles were expected specifically with BSA due to the presence of free cysteine. However, at room temperature, a significant excess of the binding constant of BSA with gold nanoparticles over lysozyme was found only at pH 7.5 (4.5 × 10^8^ M^−1^ for BSA compared to 1.6 × 10^8^ M^−1^ for lysozyme) (Figure 3a,b). At other pH values, the binding constants differed insignificantly at 25 °C. This indicates that the interaction of the protein with gold nanoparticles due to the thiol group is determined by the pH of the medium. A pronounced increase in the binding constants of BSA with gold nanoparticles was observed with increasing temperature at pH 5.0 and 12.0. At pH 12.0, the cysteine residue in BSA is in the deprotonated form (-S-) [39] and is located in a hydrophobic pocket. Apparently, increasing the temperature leads to disruption of the protein structure and exposure of cysteine on the protein surface, which increases the accessibility of the thiol group to gold nanoparticles and solvent.

In general, the authors note high binding constants of BSA with Au and Ag nanoparticles (10^6^–10^9^ M^−1^) [40,41], compared to other nanoparticles SiO_2_, TiO_2_ and ZnO (102–104 M^−1^) [42,43]. Functionalization of nanoparticles with ligands not only leads to stabilization of particles but also significantly changes their interaction with proteins. In the case of gold nanoparticles without surface groups, high binding constants are observed. In the case of IONPs, surface functionalization with sodium citrate increases the binding constant with HSA by 4 orders of magnitude (up to ~10^8^ M^−1^) compared to nanoparticles without citrate [26]. In our work, citrate-coated IONPs have binding constants close to AuNPs.

Protein binding to nanoparticles also occurs through electrostatic interactions, and the strongest interactions should occur at opposite charges of the substrates [44]. BSA is positively charged at pH 2.0–4.7, lysozyme at pH 2.0–11.0, and nanoparticles are negatively charged over the entire pH range (Table 1) [27,45]. In general, the highest binding to IONPs was observed at pH 7.5 for lysozyme and at pH 5.0 for BSA (Figure 3c,d). Moreover, the most pronounced effect of temperature on the interaction of iron oxide nanoparticles with lysozyme was observed at different pH. Previously, it was shown that iron oxide nanoparticles stabilize the structure of lysozyme with increasing temperature and pH 7.25 [46]. In our study, an increase in the association constant of lysozyme with iron oxide nanoparticles with increasing temperature was observed at pH 7.5 (Figure 3c). In the case of BSA, the highest binding was observed at pH 5.0 (the nanoparticles are negatively charged, the protein charge is almost zero). Only at this pH was a significant effect of temperature on the binding constant observed. It has previously been shown that the greatest amount of BSA protein is adsorbed onto the surface of iron oxide nanoparticles at the isoelectric point (~4.7–4.9) [47]. It is likely that in the case of BSA, the highest binding affinity for any type of nanoparticle is observed precisely at the isoelectric point.

Gold nanoparticles exhibit significantly higher quenching constants compared to iron oxide nanoparticles. However, no significant effect of pH or temperature on the quenching constant was observed upon addition of gold nanoparticles. Gold nanoparticles exhibit intense local surface plasmon resonance, which provides efficient non-radiative energy transfer from excited tryptophan [48]. This interaction mechanism likely depends weakly on specific environmental conditions. The fluorescence quenching constant of lysozyme was slightly higher only at pH 12.0 and at room temperature (Figure 5a). At highly alkaline pH values, tryptophan residues, typically located inside the molecule, move closer to the protein surface [49], which also increases the efficiency of energy transfer [50].

In the case of iron oxide nanoparticles, both environmental parameters (pH and temperature) under study influenced the quenching constant. A noticeable trend toward a decrease in the fluorescence quenching constant of lysozyme by iron nanoparticles was observed with increasing temperature at pH 5.0 and 7.5 (Figure 5c). Similar trends were observed in a study of the interaction of BSA and hydrophilic iron oxide nanoparticles with a static fluorescence quenching mechanism [25]. In contrast, an increase in the quenching constant was observed for interactions with lysozyme at pH 12.0 and with BSA at pH 2.0, 7.5, and 12.0 with increasing temperature (Figure 5c,d). This indicates a dynamic fluorescence quenching mechanism in these cases.

The fundamental difference in pH and temperature sensitivity between AuNP and IONP stems from differences in their mechanisms of protein interaction. AuNP rely primarily on specific covalent binding to the thiol groups (–SH) of cysteine (forming a strong Au–S bond), with the accessibility of the thiol groups highly dependent on protein conformation. Furthermore, protein structure is highly dependent on external conditions (pH and temperature). For IONP, electrostatic and hydrophobic interactions, which are modulated by environmental conditions, have the greatest influence.

## 4. Materials and Methods

### 4.1. Preparation of Protein Colloidal Solutions

Lyophilized lysozyme powder obtained from chicken egg white (HEWL, activity > 45,000 U/mg, GoldBio, Olivette, MO, USA) or BSA powder (Sigma-Aldrich, A7906, Burlington, MA, USA) were dissolved in deionized water (resistance 18.2 MΩ × cm) or aqueous nanoparticle colloids to a final concentration of 0.1 mg/mL.

### 4.2. Preparation of Aqueous Colloid of Gold Nanoparticles

Gold nanoparticles were synthesized by laser ablation of a high-purity solid gold target (99.99%) in deionized water (resistivity 18.2 MΩ cm) followed by laser fragmentation of the resulting colloid. The gold target was fixed to the bottom of a 30 mL glass cell filled with deionized water so that the thickness of the liquid layer above the target surface was 0.2 cm. A pulsed Nd:YAG laser NL300 (Ekspla, Vilnius, Lithuania) with parameters of 1064 nm, 4 ns, 1 kHz, 2.5 mJ was used as a radiation source. The laser beam was focused to a diameter of 200 μm and scanned the target surface using a galvanometric scanner LScanH (Ateko-TM, Moscow, Russia) equipped with an F-theta objective (focal length 90 mm). The total ablation duration was 30 min. The colloidal suspension was subsequently subjected to laser fragmentation to reduce the particle size and increase the monodispersity of particles in the solution. The fragmentation process lasted 90 min. A detailed description of this technique was given in our previous work [51]. For experimental use, the initial colloid was diluted with a protein solution and water. Calculated concentrations of nanoparticles were used to analyze the binding constant: 0.125, 0.2, 0.25, 0.3, and 0.375 nM. Where the concentration of nanoparticles is 0.125 nM, the general concentration of gold in the colloid is about 60 μM. The surface area for a 25 nm gold nanoparticle was about 1963 nm^2^. The surface area of the nanoparticle here and below was calculated using the formula for the area of a sphere.

### 4.3. Preparation of Aqueous Colloid of Iron Oxide Nanoparticles

Iron oxide nanoparticles stabilized with sodium citrate were synthesized by coprecipitation from an aqueous mixture of iron(II) and iron(III) chloride salts. A detailed synthesis protocol was described previously [52]. The prepared colloid contained nanoparticles at a concentration of 10^16^ particles/mL. For experimental use, the stock solution was diluted with a protein solution and water. Calculated concentrations of nanoparticles were used to analyze the binding constant: 0.83, 1.25, 1.66, 4.15, and 8.3 nM. Where the concentration of nanoparticles is 0.83 nM, the general concentration of iron in the colloid is about 5 μM. The surface area for a 6 nm iron oxide nanoparticle was about 113 nm^2^.

### 4.4. Transmission Electron Microscopy

A Libra 200 Fe HR transmission electron microscope (Carl Zeiss AG, Oberkohen, Germany) was used to study the morphology of the nanoparticles. Samples (0.25 µL) were applied to a gold grid with a titanium holder. The samples were dried at room temperature for 10 min and then evacuated.

### 4.5. Measurement of Optical Absorption of Colloids

Absorption spectra were measured on a Cintra 4040 spectrophotometer (GBC Scientific Equipment, Melbourne, Victoria, Australia) in quartz cuvettes with an optical path length of 1 cm. For each sample, absorption spectra were measured three times in the range of 230–400 nm with a scan rate of 900 nm/min and a slit width of 2 nm.

### 4.6. Fluorescence Spectroscopy of Colloids

Protein fluorescence in aqueous colloids was studied using a Jasco FP-8300 spectrometer (JASCO, Hachioji, Japan). Measurements were performed in a quartz cuvette with a 1 cm optical path and a 2 mL sample volume. Each sample was measured in triplicate. Fluorescence excitation for all samples was performed at a wavelength of 280 nm. Sample emission was recorded in the 295–450 nm range; the scanning rate was 1000 nm/min.

### 4.7. Changes in pH and Temperature of Colloids

The pH values of 2.0, 5.0, 7.5, and 12.0 were used based on the results of a pilot study with lysozyme and gold nanoparticles conducted previously at room temperature [45]. At pH 2.0, the proteins are in a molten globule state and are positively charged. At pH 5.0, BSA is near the isoelectric point and in its native conformation. Under these conditions and at high concentrations (~200 mg/mL), lysozyme is most stable in solution [53]. BSA exhibits the greatest stability in solution at pH 7.0 in the absence of salt ions [54]. In particular, data on the onset of lysozyme aggregate formation at pH 7.5 and a sharp increase in refractive index were obtained from [45]. pH 12.0 was chosen as the condition where both proteins in solution are negatively charged. It is worth noting that the structure of both proteins changes at extreme pH values of 2.0 and 12.0.

pH values were measured using a Seven Excellence S470 precision station (Mettler Toledo, Greifensee, Switzerland) in a 3 mL sample vial under constant stirring with a magnetic stirrer equipped with a Teflon anchor. All colloids were prepared in deionized water. The pH of the solution was adjusted by adding varying amounts of HCl (in concentration 0.1 M or 1 M for acidification) or NaOH (in concentration 0.05 M or 1 M for alkalization). The acid and alkali were added dropwise in volumes of 2–10 μL. The pH of the solution was stable for 2 h and changed insignificantly after temperature exposure.

A DC-2006 liquid thermostat (Scientz Bio, Ningbo, China) was used to heat the samples. Temperature was additionally monitored using an Aktakom ATT-2002 thermometer with a platinum electrode. Temperature stimulation was performed at 25, 45, 65, and 85 °C with incubation for 30 min. Temperature maintenance and control accuracy was ±1 °C. Temperature treatment was performed at 25, 45, 65, and 85 °C. At temperatures below 25 °C, proteins show virtually no change in their native conformation. The maximum temperature of 85 °C was chosen because most proteins undergo partial or complete denaturation at this temperature. Intervals of 20 °C are optimal because the number of temperature points selected is sufficient to track the dynamics of changes in interactions between proteins and nanoparticles.

In this study, extreme pH values (2.0 and 12.0) and extreme temperatures of up to 85 °C were selected as parameters affecting protein–nanoparticle complexes prior to their exposure to physiological conditions. We realise that such conditions do not occur in the body, except perhaps for the acidity in the stomach. However, these conditions were chosen to investigate how proteins that have lost their native conformation interact with nanoparticles.

### 4.8. Measurement of Concentration and Hydrodynamic Diameters of Nanoparticles

All measurements were performed on a Zetasizer ULTRA Red Label instrument (Malvern Panalytical Ltd., Malvern, UK). The concentration of the obtained nanoparticles was determined as particles per ml using MADLS (multi-angle dynamic light scattering) at three angles: 174.7°, 90°, and 12.78°. The concentration was converted to molarity using Avogadro’s number, without calculating the nanoparticles’ molar mass. Colloids with varying nanoparticle concentrations were obtained from the initial solution by dilution. Information on the use of DLS to determine nanoparticle concentration is provided in the works [55,56,57].

We compared the iron concentrations in the resulting nanoparticles with those in the original iron chloride salts from which they were obtained. A detailed method for producing iron oxide nanoparticles from iron chloride salts was described in a previous publication [58]. It turned out that the concentration of iron in nanoparticles is similar in order of magnitude to that calculated from salts. Thus, we consider it possible to use this approach to calculate the molar concentration of nanoparticles in our case.

Changes in hydrodynamic diameter were recorded using dynamic light scattering. For particle size measurements, scattered light was measured at an angle of 174.7°. Intensity distributions by size were calculated using ZS Xplorer software v.4.1.0. Measurements were performed on 1 mL of sample in a thermostatic mode at 25 °C. Three independent measurements were performed for each sample.

### 4.9. Measurement of the Electrokinetic Potential of Nanoparticles

Measuring electrokinetic potential is a standard procedure in nanoparticle research. Of interest is how protein binding to nanoparticles affects particle stability. It was also interesting to observe changes in the colloid’s zeta potential upon heating at different pH values. The electrokinetic potential of particles in solution was determined using a Zetasizer ULTRA Red Label analyzer (Malvern Panalytical Ltd., Malvern, UK) at 25 °C. All measurements were performed in automatic mode and with automatic attenuator selection (the number of cycles per measurement varied from 10 to 100). The interval between experiment repetitions and the equilibration time were each 100 s.

### 4.10. Calculation of the Binding Constant Using the Benesi–Hildebrand Equation

The binding constant allows one to determine the affinity of interaction of proteins with nanoparticles and can be represented as:

(1) $$K_{a}=\frac{\left[P\cdot N\right]}{\left[P\right]\cdot [N]}$$

where [*P*], [*N*], and [*P*∙*N*] are the concentrations of proteins, nanoparticles, and protein–nanoparticle complexes, respectively.

There are various methods for determining the binding constants of reagents. When choosing optical methods to describe protein–nanoparticle interactions, one of the frequently used methods of determining the binding constant through the equation based on the Benesi–Hildebrand (B–H) equation, considering that the concentration of nanoparticles is much lower than the protein concentration [59,60,61]:

(2) $$A_{obs}=\left(1-\alpha \right)C_{0}\varepsilon_{P}+\alpha C_{0}\varepsilon_{PN}$$

where *A_obs_* is the observed absorption of proteins with different nanoparticle concentrations at a wavelength of 280 nm (the absorption maximum of aromatic amino acid residues), *α* is the fraction of association between proteins and nanoparticles, *ε_P_* and *ε_PN_* are the molar extinction coefficients of the protein and the protein with nanoparticles at a wavelength of 280 nm, and *C*_0_ is the initial protein concentration. Using the Beer–Lambert law, Equation (2) can be rewritten as follows:

(3) $$A_{obs}=\left(1-\alpha \right)A_{0}+\alpha C_{0}A_{comp}$$

where *A*_0_ and *A_comp_* are the absorption of proteins and the protein–nanoparticle complex, measured at a wavelength of 280 nm. The coefficient *α* can be represented as follows:

(4) $$\alpha =\;\frac{K_{a}\cdot [N]}{{1+K}_{a}\cdot [N]}$$

then, Equation (3) can be rewritten as:

(5) $$\frac{1}{A_{obs}-A_{0}}=\frac{1}{A_{comp}-A_{0}}+\frac{1}{K_{a}\left(A_{comp}-A_{0}\right)[N]}$$

It is worth mentioning that the method has some limitations and is applied for a 1:1 reagent ratio. Despite this, the method is often used to evaluate protein binding to nanoparticles when the protein is in excess [62,63,64,65]. In this case, the protein concentration remains virtually constant. It is under these pseudo-first-order reaction conditions that the complex process of multisite protein adsorption on the nanoparticle surface can be reduced to the ligand–receptor kinetic model (1:1). This allows one to consider nanoparticle–protein binding as a reaction where the rate depends only on the nanoparticle concentration. In addition, a range of nanoparticle concentrations was chosen where the absorption changes most linearly. This made it possible to obtain reproducible binding constant values suitable for comparison in the protein–nanoparticle series. In general, in this work, the Benesi–Hildebrand method is used only as a tool for comparing the binding efficiency of, for example, two types of nanoparticles with one protein, but not for obtaining absolute values of association constants.

### 4.11. Calculation of the Quenching Constant Using the Stern–Volmer Equation

One common method for assessing nanoparticle–protein interactions in terms of binding affinity, stoichiometry, and cooperativity is the determination of the fluorescence quenching constant [22]. There are two distinct types of fluorescence quenching: static and dynamic. Static quenching is caused by the formation of a nonfluorescent complex between the fluorophore and quencher before photon absorption, whereas dynamic quenching is a diffusion process in which the collision of the fluorophore with the quencher occurs in an excited state and leads to nonradiative deactivation. Static quenching depends on the quencher concentration, while dynamic quenching depends on both concentration and temperature [66].

The fluorescence quenching process can be analyzed using the Stern–Volmer equation:

(6) $$\frac{F_{0}}{F}=1+K_{sv}[Q]$$

where *F*_0_ and *F* are the protein fluorescence intensities in the absence and presence of nanoparticles, respectively, *K_sv_* is the Stern–Volmer (SV) quenching constant, and [*Q*] is the quencher (nanoparticle) concentration in M.

When adding nanoparticles to a protein solution, the absorption of the nanoparticles at the 280 nm wavelength used should be taken into account. Particularly strong absorption is observed with a solution of iron oxide nanoparticles. This absorption of excitation or emission light is known as the inner filter effect and will be reflected in the fluorescence spectra and the calculation results of the quenching constant [67]. In a spectrofluorimeter, the center of the cuvette is the excitation point and the initial emission point. Consequently, if the absorption of the excitation light is significant, the amount of light reaching the center decreases, and the emission intensity decreases. Therefore, the attenuation of light intensity caused by the absorption of the nanoparticles must be taken into account. The fluorescence intensity was corrected using the relationship [68,69]:

(7) $$F_{corr}=F_{obs}\times e^{\frac{A_{ex}+A_{em}}{2}}$$

where *F_corr_* and *F_obs_* are the corrected and observed fluorescence intensities, respectively, and *A_ex_* and *A_em_* are the absorption at the excitation and emission wavelengths, respectively.

A necessary condition for applying the Stern–Volmer equation is a linear relationship between the change in the fluorescence of the protein solution and the concentration of nanoparticles. The addition of nanoparticles at concentrations of 0.125–0.375 nM leads to a linear decrease in protein fluorescence (see Figure 4). Studies determining the fluorescence quenching constant of proteins at much lower nanoparticle concentrations are common [70,71].

### 4.12. XRD

**Figure A9:**
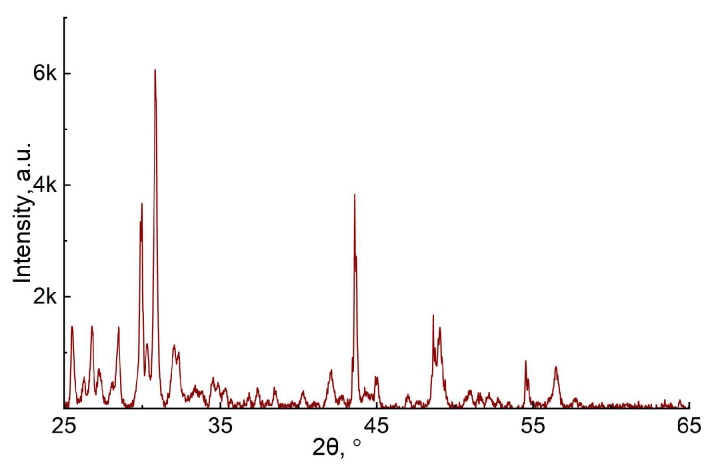
XRD diffractogram of iron oxide nanoparticle powder after drying.

X-ray diffraction was performed on a Tongda TD-3700 powder diffractometer using CuKα radiation (λ ≈ 1.5406 Å) (Dandong Tongda Science & Technology Co., Ltd., Dandong, China). The measurements were performed in Bragg–Brentano geometry with a parallel beam formed by a Göbel mirror. This setup ensures a low angular divergence of the primary beam and a constant instrumental width of diffraction peaks over the entire 2θ range (from 20° to 70°). The imaging parameters were a 0.02° step and an exposure selected for weak nanoscale phases. Primary processing (background, peak detection, indexing) was performed using the COD and CCDC databases. Full-profile analysis was performed using the Le Bail method. The following parameters were refined: phase scaling factors, unit cell parameters, profile parameters (pseudo-Voigt, U parameter for estimating microstrains/dimensions). The instrumental profile was determined using a reference silicon sample, followed by refinement of the profile function parameters. The Scherrer equation, corrected for instrumental broadening, was used to estimate crystallite sizes. The resulting XRD diffractogram is shown in Figure A9. The following crystalline phases were identified in the sample: NaCl (main), α-NH_4_Cl (main), β-NH_4_Cl (auxiliary), Na_3_(C_6_H_5_O_7_)·2H_2_O (TSC) and nanosized Fe_3_O_4_/γ-Fe_2_O_3_. Refinement quality: R_wp_ = 9.1%, R_p_ = 6.01%. Crystallite size: NaCl ~ 70 nm, NH_4_Cl ~ 40–50 nm, TSC ~ 30–40 nm, iron oxides ~ 1–7 nm. Content: salts ~ 70%, TSC ~ 20%, iron oxides <10% (semi-quantitative). The presence of a nanoscale oxide phase with a size of about 6 nm is consistent with the results obtained previously [58] and is consistent with the synthesis conditions.

## 5. Conclusions

The paper presents the results of calculating the binding and quenching constants of two model proteins with two types of nanoparticles under varying pH and temperature conditions. The selected proteins exhibit both high binding affinity to both types of nanoparticles (10^7^–10^9^ M^−1^) and high quenching constant values (10^8^–10^9^ M^−1^) in the selected pH and temperature ranges. However, the highest binding and quenching constants were observed for proteins with gold nanoparticles. At the same time, no obvious effect of pH and temperature on either the binding or quenching constants of proteins with gold nanoparticles was found for gold nanoparticles. In contrast, different temperature trends in the dependence of the binding and quenching constants on temperature and pH were observed for iron oxide nanoparticles, and these effects were different for BSA and HEWL. The association and quenching constants depended most on temperature at pH 5.0 for BSA with iron oxide nanoparticles. In the case of lysozyme, the greatest effect of temperature on association was observed at pH 7.5, and on fluorescence quenching at pH 12.0.

Overall, it seems possible to take these results into account when rationally designing biomedical systems. Protein-encapsulated gold nanoparticles represent a system with predictable behaviour, weakly dependent on specific external conditions (as no significant effects of pH and temperature on the binding and quenching constants were detected). This makes them reliable candidates, for example, for delivery systems where stability and reproducibility under various conditions are important. In contrast, protein-encapsulated iron oxide nanoparticles are highly sensitive to pH and temperature and can selectively respond to the microenvironment.

## Figures and Tables

**Table 1:** Electrokinetic (Zeta) potential values for colloids at 25 °C with concentrations of HEWL/BSA 0.1 mg/mL, AuNP (0.375 nM), IONP (4.15 nM).

Colloids	Zeta Potential, mV			
	pH 2.0	pH 5.0	pH 7.5	pH 12.0
AuNP	−12.2 ± 1.1	−26.0 ± 2.4	−31.0 ± 0.4	−29.0 ± 1.9
IONP	−6.4 ± 0.7	−23.8 ± 0.5	−27.4 ± 2.3	−35.6 ± 3.9
HEWL	+12.6 ± 0.9	+16.0 ± 0.3	+8.0 ± 1.3	−23.0 ± 1.5
BSA	+8.3 ± 0.4	−2.7 ± 0.8	−20.8 ± 1.3	−23.6 ± 1.1
HEWL+AuNP	+25 ± 2.1	+33.0 ± 0.2	+12.0 ± 0.5	−22.0 ± 1.6
HEWL+IONP	+22.0 ± 1.5	+28.0 ± 0.4	+10.0 ± 0.7	−23.0 ± 0.8
BSA+AuNP	+20.0 ± 0.4	+5.0 ± 1.2	−25.0 ± 0.8	−26.0 ± 0.9
BSA+IONP	+24.5 ± 0.2	−2.5 ± 1.0	−15.8 ± 0.6	−33.0 ± 0.4

## Data Availability

The data presented in this study are available on request from the corresponding author (Order of the Director of Federal Scientific Agroengineering Center VIM).

## Abbreviations

The following abbreviations are used in this manuscript:

BSA	Bovine Serum Albumin
AuNP	Aurum NanoParticles
IONP	Iron Oxide NanoParticles
HEWL	Hen Egg White Lysozyme

## Appendix A

**Table A1:** Association (*K_a_*) and fluorescence quenching (*K_sv_*) constants of HEWL and BSA proteins with AuNPs and IONPs under different pH and temperature conditions.

Temperature, °C	pH 2.0	pH 5.0	pH 7.5	pH 12.0
*K_a_* × 10^8^, M^−1^				
HEWL+AuNP				
25	3.21 ± 0.41	5.39 ± 0.72	1.65 ± 0.50	2.61 ± 0.26
45	2.51 ± 0.25	2.87 ± 0.47	7.57 ± 0.12	3.87 ± 0.53
65	3.45 ± 0.80	2.30 ± 0.30	7.51 ± 0.24	3.07 ± 0.63
85	1.55 ± 0.44	1.55 ± 0.44	4.35 ± 0.74	4.45 ± 0.66
HEWL+IONP				
25	1.44 ± 0.12	1.07 ± 0.09	1.41 ± 0.09	1.00 ± 0.06
45	1.29 ± 0.14	1.78 ± 0.10	1.65 ± 0.04	1.71 ± 0.16
65	0.62 ± 0.08	1.34 ± 0.20	2.35 ± 0.43	0.83 ± 0.07
85	0.47 ± 0.04	2.13 ± 0.47	9.24 ± 5.2	1.00 ± 0.25
BSA+AuNP				
25	3.81 ± 0.56	5.78 ± 0.49	4.53 ± 0.45	2.51 ± 0.33
45	4.80 ± 0.35	7.52 ± 0.91	4.63 ± 0.04	5.38 ± 0.61
65	3.60 ± 0.69	3.07 ± 0.82	13.82 ± 1.29	5.38 ± 0.71
85	6.08 ± 0.64	16.43 ± 1.57	5.44 ± 0.84	20.16 ± 2.04
BSA+IONP				
25	1.25 ± 0.10	2.31 ± 0.31	1.91 ± 0.20	1.41 ± 0.20
45	1.32 ± 0.15	2.10 ± 0.30	1.91 ± 0.17	1.59 ± 0.21
65	1.15 ± 0.20	8.65 ± 3.31	1.66 ± 0.20	1.57 ± 0.29
85	1.32 ± 0.20	0.53 ± 0.09	1.70 ± 0.26	1.62 ± 0.17
*Ksv* × 10^8^, M^−1^				
HEWL+AuNP				
25	11.28 ± 1.28	6.26 ± 2.10	8.77 ± 1.41	21.45 ± 4.80
45	9.63 ± 2.17	14.47 ± 3.07	15.73 ± 3.10	15.21 ± 3.30
65	1.38 ± 1.86	10.07 ± 1.67	8.89 ± 0.92	15.44 ± 2.60
85	9.76 ± 3.11	17.20 ± 4.71	12.37 ± 1.93	24.05 ± 3.96
HEWL+IONP				
25	1.93 ± 0.19	2.40 ± 0.21	3.49 ± 0.36	0.98 ± 0.20
45	1.10 ± 0.16	1.81 ± 0.49	2.20 ± 0.28	1.44 ± 0.11
65	1.28 ± 0.03	1.17 ± 0.24	1.65 ± 0.13	6.81 ± 0.83
85	1.73 ± 0.36	0.94 ± 0.15	1.71 ± 0.43	3.65 ± 0.57
BSA+AuNP				
25	14.28 ± 1.54	6.92 ± 1.13	7.50 ± 0.53	7.33 ± 1.90
45	11.38 ± 1.92	6.97 ± 0.96	6.31 ± 0.65	9.93 ± 2.14
65	13.27 ± 0.76	14.48 ± 4.01	12.08 ± 2.30	7.08 ± 1.70
85	15.31 ± 1.40	19.74 ± 3.08	12.43 ± 0.62	12.83 ± 1.55
BSA+IONP				
25	1.44 ± 0.05	15.40 ± 1.01	2.49 ± 0.27	1.45 ± 0.16
45	1.64 ± 0.10	12.88 ± 0.88	2.36 ± 0.31	1.41 ± 0.17
65	1.70 ± 0.16	15.88 ± 1.50	3.07 ± 0.18	2.13 ± 0.19
85	2.46 ± 0.07	6.94 ± 0.84	4.30 ± 0.31	2.72 ± 0.23
